# Establishment and Characterization of a New Cell Line from Enzootic Nasal Adenocarcinoma in Goats: ENA-1

**DOI:** 10.3390/vetsci11060260

**Published:** 2024-06-07

**Authors:** Lingxu Li, Weiye Tan, Zhen Wang, Wenqing Guo, Deji Yang, Dawei Yao

**Affiliations:** College of Veterinary Medicine, Nanjing Agricultural University, Nanjing 210095, China; 2023207040@stu.njau.edu.cn (L.L.); 2022807214@stu.njau.edu.cn (W.T.); 2022107022@stu.njau.edu.cn (Z.W.); 2023807235@stu.njau.edu.cn (W.G.); djyang@njau.edu.cn (D.Y.)

**Keywords:** cell line, enzootic nasal tumor virus, goat, enzootic nasal adenocarcinoma

## Abstract

**Simple Summary:**

Enzootic nasal adenocarcinoma is an infectious, chronic tumor disease of goats and sheep. Tumor cell lines are very important tools in tumor research. This manuscript describes the isolation, culture, and characterization of a new enzootic nasal adenocarcinoma cell line from goats. This is the first report about an enzootic nasal adenocarcinoma cell line. ENA-1 cells are stable in passaging, have strong proliferative potential and tumorigenicity in nude mice, express primitive tumor phenotypes, and carry enzootic nasal tumor virus.

**Abstract:**

Enzootic nasal adenocarcinoma (ENA) is a contagious tumor disease of goats and sheep, which is caused by enzootic nasal tumor virus (ENTV). To better understand the pathogenesis of ENA, this study aimed to establish a goat ENA cell line (ENA-1). The cells have been characterized with regard to morphology, growth rate, ultrastructural features, chromosome number, expression of CK7 and CK18, tumorigenicity, species, and mycoplasma contamination. ENA-1 had an epithelioid cell morphology with an unstable chromosome number under a light microscope. Under an electron microscope, the cell nuclear heterogeneity was not obvious, and there were more intermediate filaments and a small number of immature retrovirus-like particles in the cytoplasm. ENA-1 had strong proliferative potential, and the cell multiplication time was about 36 h, which could make BALB/c nude mice develop tumors. CK7 and CK18 were expressed in the cytoplasm of primary goat tumors, in transplanted tumors from nude mice, and un ENA-1 cells with the same intensity. PCR revealed that ENA-1 continuously carried ENTV-2 up to the 17th generation with no germline contamination or mycoplasma contamination. In conclusion, using a serum-containing culture system, ENA-1 cells were successfully isolated, cultured, and purified from goat tumor tissues. The isolated ENA-1 cells retained robust proliferation potential and maintained their phenotype, indicating the potential application of the ENA-1 cell line as an in vitro model of ENA.

## 1. Introduction

Enzootic nasal adenocarcinoma (ENA) is an infectious, chronic tumor disease of goats and sheep, which is caused by enzootic nasal tumor virus (ENTV) [1]. In China, it has been reported nationwide and has been expanding in recent years, causing large economic losses to the goat farming industry [2,3,4]. Tumors grow in the nasal cavity of diseased goats, and as the size of the tumor increases, they show clinical symptoms such as the flow of plasma nasal fluid and heavy breathing sounds [5]. The disease has a long course, with a low incidence but a 100% mortality rate, and there is no vaccine or drug to prevent or control it [6].

Tumor cell lines are crucial tools in tumor research that facilitate the study of tumorigenesis mechanisms and the screening of therapeutic drugs. However, there are limited studies on ENA tumor cell lines. To date, only one study has reported the isolation and culture of an ENA cell line from sheep, which has undergone more than 30 passages and in which exogenous ENTV-1 was detected by PCR in early culture [7]. There has been no comprehensive study reporting the purification and characterization of a goat ENA cell line. Therefore, our study aims to establish a stable passaging, morphologically homogeneous goat ENA cell line. This process will involve isolating, culturing, and thoroughly characterizing the goat ENA cell line. Achieving our goal will provide a valuable model for studying the disease and will potentially aid in the development of new therapeutic strategies.

## 2. Materials and Methods

### 2.1. ENA Tumor Tissues

A female Anhui white goat, a Chinese indigenous breed, became sick at a farm. Clinical manifestations of this goat included heavy respiratory sounds, nasal discharge, and reddening of the skin around the nostrils. ENA was confirmed by autopsy, histopathological examination, and PCR method. The tumor was pale white or pink, cauliflower-shaped, and was located in the right nasal cavity. The tumor was closely connected with the ethmoidal labyrinth and extended to the front of the nasal cavity along the turbinate bone. The ethmoid bone and turbinate bone in the right nasal cavity were damaged and extruded. In histopathological sections of tumor tissues, the ENA was an adenocarcinoma with predominantly well-differentiated regions and some poorly differentiated regions. Papillae and acini were located in the superficial and deeper layers of the tumor, respectively. Mitotic figures were rarely observed. There was no apparent tumor infiltration in the vessels and no necrotic areas. Nasal secretion samples were collected from goats after death, RNA was extracted, primers were designed according to published research [8], and the samples had the target bands assessed by RT-PCR.

Tumor tissues were collected to establish cell line models after death. The protocol and procedures employed in this study were ethically reviewed and approved by the ethics committee at Nanjing Agricultural University.

### 2.2. Cell Line Establishment

Tumor tissues were washed three times with phosphate-buffered saline (PBS, P1020, Solarbio, Beijing, China) with 100 U/mL penicillin, 0.1 mg/mL streptomycin, and 0.2 mg/mL ceftiofur sodium. Tumor tissues were finely minced (1 mm^3^) using a scalpel blade in a petri dish and transferred to a 25 cm^2^ cell culture flasks. Twenty tumor tissues pieces were transferred to each cell culture flask and cultured in complete RPMI-1640 medium (11875, Solarbio, Beijing, China) supplemented with 10% fetal bovine serum (FB25015, Clark Bioscience, Richmond, VA, USA), 100 U/mL penicillin, 0.1 mg/mL streptomycin, and 0.2 mg/mL ceftiofur sodium. The tumor tissues pieces were routinely cultured in an incubator at 37 °C in 5% carbon dioxide. The media was replaced with fresh complete RPMI-1640 medium once every 3 days. The cell status was observed every day.

At 90% confluency, the cells were washed twice with phosphate buffer (D-PBS, D1040, Solarbio, Beijing, China) and were detached with 1 mL 0.25% trypsin (T1350, Solarbio, Beijing, China) for 5 min at 37 °C and then gently resuspended in 5 mL complete RPMI-1640 medium. The cells were centrifuged at 1200 r/min for 5 min and resuspended in 1 mL complete RPMI-1640 medium. The cells (2 × 10^5^ cells/mL) were seeded in 25 cm^2^ cell culture flasks with 5 mL complete RPMI-1640 medium.

### 2.3. Single Cell Cloning

Cells at the 5th passage were trypsinized and resuspended using complete RPMI-1640 medium. The cells were diluted to 100 cells in 10 mL, and 100 µL was pipetted into each well of a 96-well plate. The wells with one colony only had circles draw around them under an inverted microscope. The number of colonies and colony sizes were evaluated on the third day and then fresh complete RPMI-1640 medium was added. Once the clones had expanded to 50% confluency, they could be passaged to a 24-well plate to expand cultivation. This cell line was named ENA-1.

### 2.4. Electron Microscopy of ENA-1

ENA-1 cells were seeded into 10 cm^2^ dishes, and the medium was aspirated when it reached about 80% confluence. The cell monolayers were washed with PBS. After the cells were washed three times with 1 × PBS, they were scraped off with a cell scraper and collected in 1.5 mL tubes with centrifugation at 1800 r/min for 10 min. Then, 1 mL 2.5% glutaraldehyde was slowly added to the tube along the tube wall. Then, the cells were dehydrated in a 50–100% ethanol gradient and embedded in araldite. Ultrathin sections were obtained and stained with uranyl acetate and lead citrate. Sections were viewed with a transmission electron microscope (H-7650, Hitachi, Ltd., Tokyo, Japan).

### 2.5. Cell-Growth Kinetics of ENA-1

To investigate ENA-1 cell-growth kinetics, the cell suspension was diluted to 1.5 × 10^4^ cells per mL and 1 mL of cell suspension was plated into each well of a 24-well plate. Three parallel well plates were set for each group. Cells were counted in triplicate at time intervals of 24 h for 10 consecutive days. Cell-population-doubling time was determined using the equation TD = lg2/((lgNt − lgN0)/∆t), where TD is cell-population doubling, Nt is cell number at time t, N0 is cell number at initial culture, and ∆t is the time in days.

### 2.6. Karyotype Analysis of ENA-1

When cells were in the late log phase of growth, colchicine (final concentration of 0.2 μg/mL) was added to the medium. The cells were washed once with PBS buffer and routinely digested and collected into centrifuge tube. The cells were evenly suspended with hypotonic KCl (0.075 mol/L) in a 37 °C water bath for 20 min. Then the cells were centrifuged at 1000 r/min for 5 min and fixed in a chilled, freshly prepared mixture of acetic acid:ethanol (1:3) at room temperature. Centrifugation and fixing steps were repeated three times. Cell suspensions were dropped onto ethanol-cleaned and pre-cooled glass slides and immediately heat fixed by quickly passing them over a flame and staining them with freshly prepared Giemsa stain. The slide was air dried, then checked under the microscope. Cells with intact morphology, well-dispersed chromosomes, and a clear boundary in metaphase were selected. The chromosome number was determined from 50 mitotic cells.

### 2.7. Immunofluorescence Staining of ENA-1 Cells 

In a 12-well plate, sterilized cover slides were placed in each well. Cells were seeded in a 12-well plate with 4 × 10^4^ cells/well and grown to 60% confluency. Cells were washed twice with PBS, fixed with 4% paraformaldehyde for 20 min, washed again, permeabilized with 0.3% Triton-X 100 for 30 min, and blocked with 3% bovine serum albumin for 30 min. Slides were incubated with primary antibodies against Cytokeratin 7 (CK7, 1:150, DF7027, Affinity Biosciences, Cincinnati, OH, USA) and Cytokeratin 18 (CK18, 1:150, AF0191, Affinity Biosciences, Cincinnati, OH, USA) diluted in blocking solution overnight at 4 °C. Primary antibodies were human-specific antibodies against a synthesized peptide of CK7 and CK18. After 3 washes in PBS, the slides were incubated with a fluorescent secondary antibody for 1 h at room temperature then washed 3 times. The slides were observed under a fluorescence microscope. PBS was used instead of the primary antibody to serve as a blank control.

### 2.8. In Vivo Tumor Xenograft Experiments with ENA-1

Ten 4-week-old female and male immunodeficient BALB/c nude mice (Beijing Vital River Laboratory Animal Technology Co., Ltd., Beijing, China) were housed in the Barrier Environment at the Laboratory Animal Center of Nanjing Agricultural University. A 0.2 mL volume of tumor cell suspension (1 × 10^7^ cells/ml) was subcutaneously injected into each mouse at the left under-axillary for tumor implantation. The nude mice were raised for 4 weeks and sacrificed after anesthesia.

The xenograft tumor was excised and fixed in 10% neutral buffered formalin for histopathological and immunohistochemistry examination. Immunohistochemistry was performed using CK7 antibody (1:100, DF7027, Affinity Biosciences, Cincinnati, OH, USA), CK18 antibody (1:100, AF0191, Affinity Biosciences, Cincinnati, OH, USA), and the Super -maxvision mouse/rabbit universal HRP Kit (1:200, TPB-0015, Typing Biotech Co., Ltd. of Nanjing, China) by routine methods and results were observed with a conventional optical microscope. PBS was used instead of the primary antibody to serve as a blank control. All animal experiments met ethical standards and were approved by the ethics committee at Nanjing Agricultural University (NJAU.No20221101210).

### 2.9. ENTV-2 Detection of ENA-1

ENA-1 cells were passaged to the 30th generation and each generation of the cells was collected for ENTV-2 detection. The cell samples were washed 3 times with 1 × PBS, and 1 mL RNAiso Plus lysate was added. Total RNA was extracted from cell samples and culture medium samples from each generation. ENTV-2 were detected by a PCR method as described by Li et al. [8].

### 2.10. Authentication of ENA-1

The cells were centrifuged by conventional trypsinization and resuspended in DNA lysate. DNA extraction was performed by the phenol-chloroform method, and cell line species were identified by PCR assay. Specific primers were designed and selected based on differences in microsatellite genomes and the cytochrome C oxidase I (COI) genes of diverse species (Table 1). The PCR volume was 25 μL, containing 12.5 μL 2 × Es Taq MasterMix (CWBIO, Beijing, China), 1 μL each of the primers, 2 μL template, and 8.5 μL double distilled water. The cycling conditions were as follows: 94 °C for 5 min; 30 cycles of 94 °C for 15 s, 60 °C for 15 s and 72 °C for 40 s; and a final extension cycle of 10 min at 72 °C. The products were examined by 1.5% agarose gel electrophoresis.

### 2.11. Mycoplasma-Contamination Detection

The previously described PCR method was used for the verification of mycoplasma contamination. Cell culture supernatants (100 μL) were collected for DNA extraction by the phenol-chloroform method. A pair of previously reported primers (PPLO-F: 5′-TTACGCAAGAGAATGCTTCA-3′, PPLO-R: 5′-TAGGAAA GCACCCTATTGAT-3′) was used to detect mycoplasma (1626 bp) in samples [11]. The PCR reaction contents and conditions were the same as above. The annealing temperature was 58 °C and the annealing time was 2 min. A laboratory-retained *Mycoplasma ovipneumoniae* standard strain was used as a positive control.

## 3. Results

### 3.1. Microscopic and Ultrastructural Morphology of ENA-1 Cells and Growth Assay

**Figure 1:**
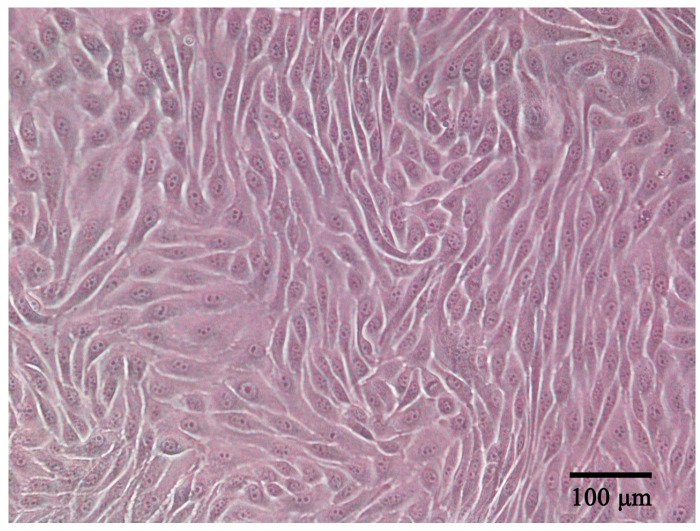
ENA-1 cells exhibited an epithelial-like morphology under the microscope (bar—100 μm).

ENA-1 cells had an epithelial-like appearance. The cells contained abundant cytoplasm and a large nucleus; showed adherent growth; and exhibited triangular, fusiform, ovoid, or polygonal shapes (Figure 1). When the cells reached a density of 90%, cells were stacked on top of each other in some regions, which is a characteristic of cells that have lost contact inhibition. The cells were stably propagated over 40 passages. The cell line was deposited in the China Center for Type Culture Collection (CCTCC) with accession number C2022232.

**Figure 2:**
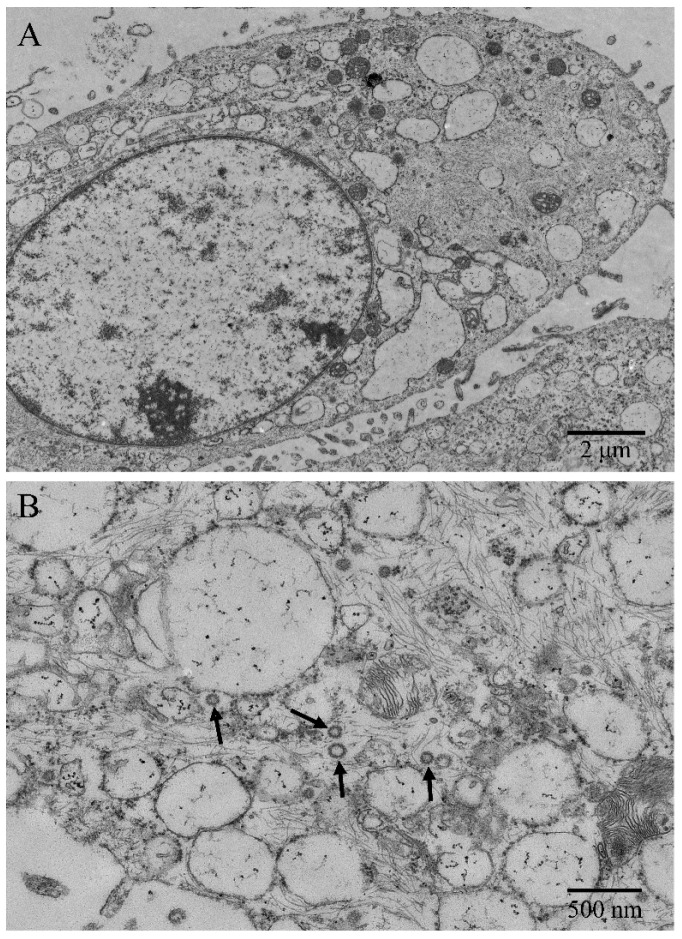
Ultrastructural morphology of ENA-1 cells. (**A**) The cells had large numbers of microvilli-like structures, high nucleoplasmic ratio, bright nuclei, and dilated endoplasmic reticulums (bar—2 μm). (**B**): Immature ENTV-2 viral particles and intermediate filaments were visualized in the cytoplasm (black arrow; bar—500 nm).

Under the transmission electron microscope, microvilli-like structures were observed on cell surfaces, and desmosomes and tight junctions between cells were found (Figure 2A). The ENA-1 cells had a clear outline, and the shapes were mostly round, spindle-shaped, and elliptical. The cells showed high nuclear–cytoplasmic ratio but regular and bright nuclei without atypia (Figure 2A). The intermediate filaments were distributed as a fine meshwork throughout the cytoplasm. The rough endoplasmic reticulum and mitochondrial cristae were dilated. Immature ENTV-2 viral particles, sized about 80–120 nm, were visualized in the cytoplasm, showing an envelope around the particles typical of retrovirus (Figure 2B).

**Figure 3:**
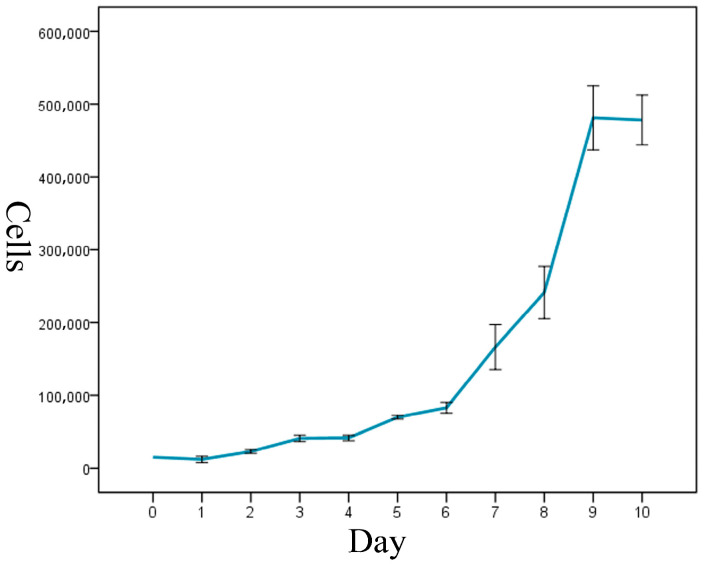
Growth curve of ENA-1 cells showed a typical ‘S’ shape with three growth stages: latency, logarithmic-growth period and plateau.

The cell-growth curve had a typical ‘S’ shape, and it displayed three growth stages: latency, logarithmic-growth period, and plateau (Figure 3). The calculated cell-doubling time of the ENA-1 cell line was approximately 36 h.

### 3.2. Karyotype Analysis

**Figure 4:**
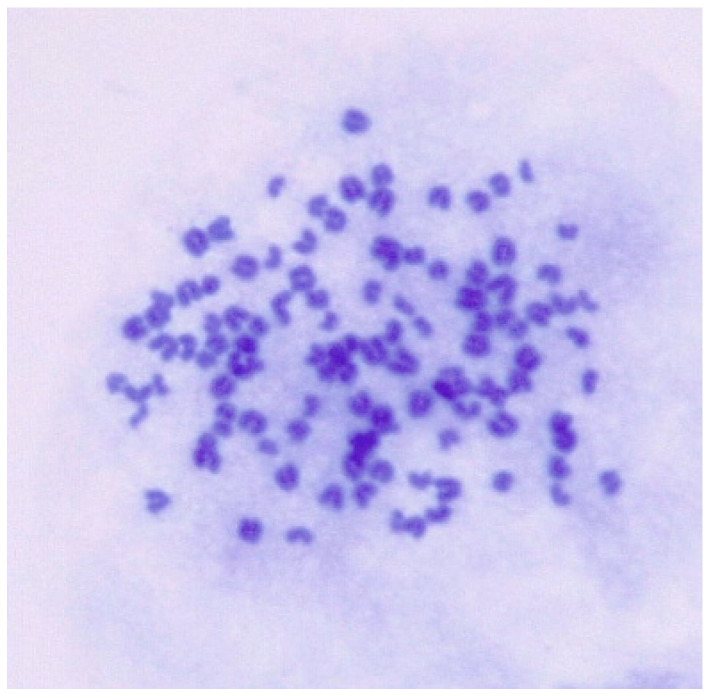
Karyotypic analysis of numerical chromosomal abnormalities in ENA-1 cells. This cell has 113 chromosomes (magnification 1000×).

The goat chromosome number was 2n = 38. However, the chromosome number for ENA-1 cell ranged from 51 to 116 and predominantly from 80 to 112, and karyotypes ranged from hypodiploid to tetraploid (Figure 4).

### 3.3. Immunocytochemical Identification of ENA-1 Cells

**Figure 5:**
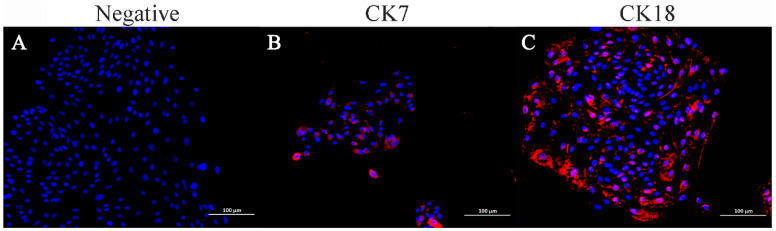
Immunofluorescence staining of ENA-1 cells for CK7 and CK18. ENA-1 cells expressed CK7 and CK18 in the cytoplasm, with CK18 most strongly expressed. (**A**) Negative control; (**B**) ENA-1 cells expressing CK7; and (**C**) ENA-1 cells strongly expressing CK18. (**A**–**C**), bar—100 μm.

Immunofluorescence staining indicated that ENA-1 cells expressed CK7 and CK18 in the cytoplasm, with CK18 most strongly expressed (Figure 5). The negative control did not express CK7 and CK18.

### 3.4. Histopathological and Immunohistochemical Characterization of the Primary Tumor in ENA Goats and Xenograft Tumors in Nude Mice

**Figure 6:**
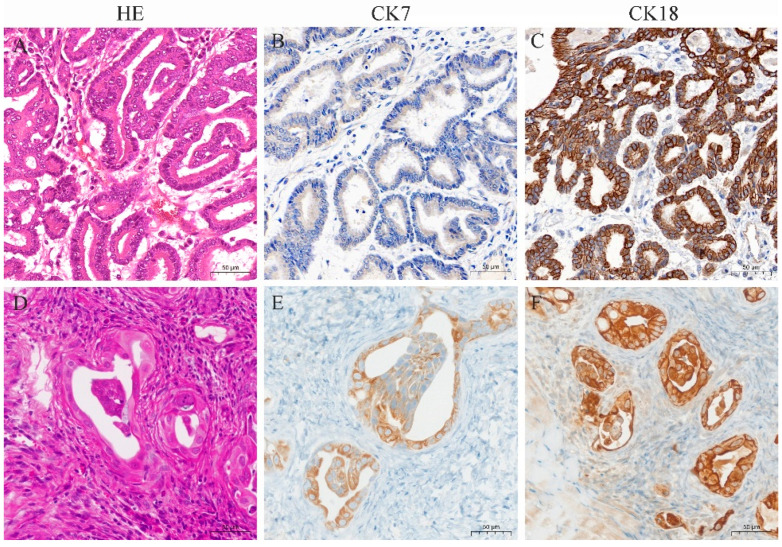
HE and immunohistochemistry for CK7 and CK18 in ENA tumors from goats and xenograft tumors from nude mice. (**A**) HE staining of ENA goat tumor with obvious highly differentiated glandular structure; (**B**) CK7 was expressed in the ENA goat tumor cytoplasm with moderate intensity; (**C**) CK18 was expressed in the ENA goat tumor cytoplasm with strong intensity; (**D**) HE staining of nude mice xenograft tumor, which formed a glandular structure with capsule; (**E**) CK7 was expressed in the nude mice xenograft tumor cytoplasm with moderate intensity; (**F**) and CK18 was expressed in the nude mice xenograft tumor cytoplasm with strong intensity. (**A**–**F**), bar—50 μm.

Histopathology of goat ENA tumors showed that the tumor types were classified as papillary and acinar. The tumors were covered with pseudostratified ciliated columnar epithelium, and the interior was dominated by highly differentiated, acini-like structures (Figure 6A). Mitotic figures were rarely seen. Immunohistochemistry showed that tumor cells diffusely and strongly expressed CK18, and diffusely and moderately expressed CK7 (Figure 6B,C).

Xenograft tumors from nude mice showed a tumor capsule that was composed of multiple layers of connective tissue. Tumor cells were highly differentiated, with distinct cell borders, glandular structures, and eosinophilic or neutrophilic cytoplasm stain (Figure 6D). The nuclei were round or oval, with light and uneven staining, located in the center or on one side of the cell. Mitotic figures were infrequent. Immunohistochemical results showed that the cytoplasm of the xenograft tumors expressed CK7 and CK18, with stronger expression of CK18 (Figure 6E,F).

### 3.5. ENTV-2-Carrying Generations

Medium samples and cell layer samples from primary cells up to and including passage 17 were positive for ENTV-2 by PCR test, and in samples from passage 18 and above cells were negative for ENTV-2.

### 3.6. Cell Species and Mycoplasma Contamination

**Figure 7:**
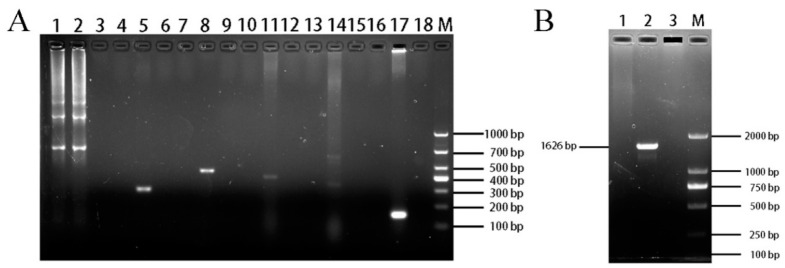
Identification of ENA-1 cells species and mycoplasma contamination. (**A)** Results of ENA-1 cells species testing. ENA-1 cells were devoid of bands for all species except for one 679 bp band for goat. Lanes 1, 4, 7, 10, 13, and 16 contained ENA-1 cells samples; lanes 2, 5, 8, 11, 14, and 17 contained positive controls for goat, chicken, canine, human, bovine, and mouse, respectively; lanes 3, 5, 9, 12, 15, and 18 contained blank controls; M—DL 2000 DNA Marker. (**B**) Results of mycoplasma-contamination testing. ENA-1 medium with no bands of mycoplasma. Lane 1, ENA-1 medium; Lane 2, Mycoplasma positive control; Lane 3, blank control; M—DL 2000 DNA Marker.

The PCR results for species testing showed that ENA-1 cells were devoid of bands in all species except for one 679 bp band for goat (Figure 7A). It indicated that the source species of the ENA-1 cells was goat and there was no interspecies contamination.

The PCR results for mycoplasma showed that ENA-1 medium gave no bands (Figure 7B), indicating that there was no mycoplasma contamination of ENA-1 cells.

## 4. Discussion

In this study, we report the isolation and characterization of the enzootic nasal adenocarcinoma cell line ENA-1. The ENA-1 cells developed in this study provide an invaluable tool for further studies on transformation mechanisms of epithelial cells and elucidating enzootic nasal adenocarcinoma tumorigenesis in response to retrovirus infection. 

ENA tumor cells are generally able to grow out of the tissue mass in about 3 days. The initial five generations of cells were more heterogeneous, with a higher percentage of fibroblast-like cells, and the fibroblast-like cells decreased significantly after five generations, whereas the growth of epithelial-like cells did not change significantly, so it was appropriate to carry out monoclonal culture of the cells after five generations. ENA-1 has a typical epithelioid cell morphology and maintains a strong proliferative capacity. The morphology of ENA-1 observed under light and electron microscopes is consistent with the morphology of epithelial cells. When the cell growth reaches the plateau stage, the cells can be observed under the light microscope to be clearly stacked, to lose contact inhibition, and to have the characteristics of tumor cells. When the ENA-1 cell line was passed on for more than 30 generations, the cell morphology did not change significantly under the light microscope, and the cell proliferation rate did not slow down significantly. 

The observation of viral particles in the ENA-1 cell line under electron microscopy was significant as it confirmed active viral infection [12]. It demonstrated that ENTV-2 was replicating within these cells. The presence of abundant intermediate filaments in the ENA-1 cell line suggested enhanced structural integrity that is crucial for survival. Intermediate filaments indicated cells in a state of rapid proliferation and some differentiation [13]. Some proteins like keratin reflected epithelial origins. Additionally, intermediate filaments are linked to cell motility and invasion [13,14]. Observing the type and quantity of intermediate filaments could help with inferring the tumor cells’ origin and behavior.

Chromosomal instability in tumor cell lines stems from cell cycle dysregulation, spindle apparatus abnormalities, high telomerase activity, defective DNA repair mechanisms, aneuploidy, and gene mutations [15]. Additionally, DNA repair deficiencies and aneuploidy increase susceptibility to chromosomal breaks. Mutations or epigenetic changes in key genes disrupt chromosomal stability [16]. These factors may lead to the chromosomal instability observed in ENA-1 cell lines.

Currently, there is no more mature method for ENA cell identification. Cytokeratin is an important marker for identifying epithelial-derived cells. CK7 and CK18 are currently commonly used to study the identification of epithelial cells of glandular origin [17,18]. Immunohistochemical staining of tumor tissue blocks was found to express CK7 and CK18 in one of our previous studies (unpublished). Therefore, positive expression of CK7 and CK18 was used as the criteria for ENA-cell identification in this study. In this study, the ENA-1 cell line and goat tumor tissues expressed CK7 and CK18, indicating that ENA-1 cells originated from tumor tissues. There is still a gap in the research into the tumorigenicity of ENA nude mice. Our established ENA-1 cell line can cause subcutaneous tumor formation in nude mice, and histologically resembles the morphology of the original tumors, both of which form a glandular cavity-like structure and express both CK7 and CK18, further indicating that ENA-1 cells originate from tumor tissues.

The ENA-1 cell line is capable of harboring ENTV-2 to the 17th generation. A study of sheep ENA cell lines showed that exogenous ENTV-1 could be detected by semi-nested PCR in four of nine isolated cell lines [7]. However, the study did not specify the generation of cells. ENTV-2 may become undetectable due to several factors: mutation or loss of parts of the viral genome, culture conditions in vitro favoring non-virus-carrying cells, suppression of viral replication, or the virus entering a latent state where it integrates into the host genome but remains transcriptionally inactive [12]. Additionally, the sensitivity of PCR may not be sufficient to detect low levels of viral nucleic acids, particularly if the virus is in a latent phase [19]. In this study, we found that ENTV-2 nucleic acid could be continuously detected in cell lines that were up to 17 generations old, and immature viral particles were also observed in the cytoplasm under electron microscopy.

## 5. Conclusions

In conclusion, ENA-1 cells were successfully isolated, cultured, and purified to create the ENA-1 cell line from ENA goat tumor tissues, which is stable in passaging, has typical morphological features of epithelial type tumors, has strong proliferative potential and tumorigenicity in nude mice and expresses primitive tumor phenotypes, and carries ENTV-2 continuously. These findings highlight the utility of the ENA-1 cell line as an in vitro model for studying goat ENA, providing valuable insights into tumor biology and viral interactions.

## Figures and Tables

**Table 1:** Primer information.

Species	Primers	Size	Reference
Bovine	actacaatccacggggtcaca	178 bp	[9]
	tgggtttcactctgggtcaat		
Chicken	taaataaaggtgttggcagtt	280 bp	[9]
	cagattgttaaaatagttgggtt		
Dog	aataatgaatgtctactttcgatgt	437 bp	[9]
	actgtgatttttgagaagagggt		
Mouse	attacagccgtacgctcctat	150 bp	[10]
	cccaaagaatcagaacagatgc		
Goat	tccgtgggtgcctagaaagtgtg	679 bp	[9]
	cctctgggagtgtggggatgca		
Human	tagacatcgtactacacgacacg	391 bp	[10]
	tccaggtttatggagggttc		

## Data Availability

The files containing the data supporting our findings can be requested directly from the corresponding author.
